# Supplementary material for: Neuro-Urology and Biobanking: An Integrated Approach for Advancing Research and Improving Patient Care
Source: Int J Mol Sci. 2023 Sep 19;24(18):14281. doi: 10.3390/ijms241814281 (PMC10531693; doi:10.3390/ijms241814281)
Supplement: Supplementary file 1 [file ijms-24-14281-s001.zip › ijms-2584108-supplementary.pdf]

SUPPLEMENTARY DATA

A systematic search was performed using the Pubmed database (www.pubmed.gov) to answer the following research questions:

Over the last 10 years (Jan 2012- Dec 2022):

1. How many studies were published in the neuro-urological research field where biobanking had been part of the study?
2. How many studies were published in which both biobanking was undertaken and neuro-urological aspects were investigated in the following relevant disease areas: Alzheimer's Disease, Spinal Cord Injury, Multiple Sclerosis, Parkinson's Disease, Stroke, Diabetes or Spina Bifida?

Methods:
A search was performed using the "advanced" function at www.pubmed.gov on August 11, 2023.
A species restriction was applied to humans.
A publication date range restriction was applied from 1.1.2012 to 31.12.2022.

Searches included terms related to the topics "biobanking/biomarkers" and "neuro-urology" which were then combined with terms specific to the diseases: Alzheimer's Disease, Spinal Cord Injury, Multiple Sclerosis, Parkinson's Disease, Stroke, Diabetes, and Spina Bifida. In total, 11 separate advanced searches were performed.

SEARCH 1: "Biobank terms"

(Biobank[Title/Abstract]
OR Biobanking[Title/Abstract]
OR "Biological resource center"[Title/Abstract]
OR "Biological sample repository"[Title/Abstract]
OR "Specimen bank"[Title/Abstract]
OR "Tissue bank"[Title/Abstract]
OR Biorepository[Title/Abstract]
OR Biocollection[Title/Abstract]
OR "Biospecimen repository"[Title/Abstract]
OR "Bioresource center"[Title/Abstract]
OR "Biomedical repository"[Title/Abstract]
OR "Human specimen bank"[Title/Abstract]
OR "Biological sample storage"[Title/Abstract]
OR "Specimen preservation"[Title/Abstract]
OR "Biorepository management"[Title/Abstract]
OR "Biospecimen banking"[Title/Abstract]
OR "Biocollection management"[Title/Abstract]
OR "Sample archive"[Title/Abstract]
OR "Tissue banking"[Title/Abstract]
OR "Specimen processing and storage"[Title/Abstract]
OR "Human sample bank"[Title/Abstract]

OR "Genetic bank"[Title/Abstract]
OR "Cell bank"[Title/Abstract]
OR "DNA bank"[Title/Abstract]
OR "Blood bank"[Title/Abstract]
OR "Organ bank"[Title/Abstract]
OR "Biomaterial bank"
OR" Living biobank"[Title/Abstract]
OR "Cryobank"[Title/Abstract]
OR "Biospecimen handling"[Title/Abstract]
OR "Biobank operations"[Title/Abstract]
OR "Biobank administration"[Title/Abstract]
OR "Specimen bank"[Title/Abstract]
OR "Sample repository"[Title/Abstract]
OR "Biospecimen repository"[Title/Abstract]
OR Samples[Title/Abstract]
OR "Sample collection"[Title/Abstract]
OR "Biomarkers"[Title/Abstract]
OR "Markers"[Title/Abstract]
OR "Tissue collection"[Title/Abstract]
OR "Tissue"[Title/Abstract]
OR "Tissues"[Title/Abstract]
OR "Biological Specimen Banks"[Mesh]
OR "Biomarkers"[Mesh])
AND ((humans[Filter]) AND (2012/1/1:2022/12/31[pdat]))

SEARCH 2: "Neuro-urology terms"

This search was split into three separate search strategies, either including neoplasms/cancer and generic terms not pointing directly to the neuro-urological field (search 2A, indicated in green below in Table S.1), or excluding neoplasms but including generic terms not pointing directly to the neuro-urological field (search 2B, in yellow), or excluding both neoplasms/cancer and generic terms not pointing directly to the neuro-urological field (search 2C, in blue).

Table S.1. Search terms related to neuro-urology, divided into three different search strategies (SEARCH 2A, 2B, 2C)

<table>
<tr><td rowspan="34">SEARCH 2A</td><td rowspan="31">SEARCH 2B</td><td rowspan="26">SEACRH 2C</td><td>"Neurogenic urology"[Title/Abstract]</td><td rowspan="26">Neuro-urology terms</td></tr>
<tr><td>OR "Neurogenic bladder"[Title/Abstract]</td></tr>
<tr><td>OR "Neurogenic voiding dysfunction"[Title/Abstract]</td></tr>
<tr><td>OR "Neurogenic lower urinary tract dysfunction"[Title/Abstract]</td></tr>
<tr><td>OR "Lower urinary tract"[Title/Abstract]</td></tr>
<tr><td>OR "Neurogenic incontinence"[Title/Abstract]</td></tr>
<tr><td>OR "Neuro-urodynamics"[Title/Abstract]</td></tr>
<tr><td>OR "Urodynamics in neurology"[Title/Abstract]</td></tr>
<tr><td>OR "Neurological urology"[Title/Abstract]</td></tr>
<tr><td>OR "Neurogenic urinary disorders"[Title/Abstract]</td></tr>
<tr><td>OR "Neurogenic urological conditions"[Title/Abstract]</td></tr>
<tr><td>OR "Neurogenic voiding disorders"[Title/Abstract]</td></tr>
<tr><td>OR "Neuro-urological conditions"[Title/Abstract]</td></tr>
<tr><td>OR "Neuro-urologic disorders"[Title/Abstract]</td></tr>
<tr><td>OR "Neuromuscular urology"[Title/Abstract]</td></tr>
<tr><td>OR "Uro-neurology"[Title/Abstract]</td></tr>
<tr><td>OR "Neurogenic bladder dysfunction"[Title/Abstract]</td></tr>
<tr><td>OR "Neurogenic lower urinary tract disorders"[Title/Abstract]</td></tr>
<tr><td>OR "Neurogenic urinary tract dysfunction"[Title/Abstract]</td></tr>
<tr><td>OR "Neuro-urological diagnostics"[Title/Abstract]</td></tr>
<tr><td>OR "Lower Urinary Tract Symptoms"[Mesh]</td></tr>
<tr><td>OR "Urinary Incontinence, Urge"[Mesh]</td></tr>
<tr><td>OR "Urinary Bladder, Overactive"[Mesh]</td></tr>
<tr><td>OR "Urinary Bladder, Underactive"[Mesh]</td></tr>
<tr><td>OR "Urinary Incontinence"[Mesh]</td></tr>
<tr><td>OR "Urinary Bladder, Neurogenic"[Mesh]</td></tr>
<tr><td rowspan="8"></td><td>OR "Urinary Tract"[Mesh]</td><td rowspan="5">Generic urology terms</td></tr>
<tr><td>OR "Urinary Retention"[Mesh]</td></tr>
<tr><td>OR "Urinary Bladder Diseases"[Mesh]</td></tr>
<tr><td>OR "Urinary Bladder"[Mesh]</td></tr>
<tr><td>OR "Urologic Diseases"[Mesh]</td></tr>
<tr><td rowspan="3"></td><td>OR "Urinary Bladder Neoplasms"[Mesh]</td><td rowspan="3">Neoplasms/cancer terms</td></tr>
<tr><td>OR "Urologic Neoplasms"[Mesh]</td></tr>
<tr><td>OR "Urogenital Neoplasms"[Mesh]</td></tr>
<tr><td></td><td></td><td></td><td colspan="2">AND ((humans[Filter]) AND (2012/1/1:2022/12/31[pdat]))</td></tr>
</table>

SEARCH 1 and SEARCH 2 (either 2A, 2B or 2C) were then combined with terms addressing the following relevant diseases:

SEARCH 3 "Alzheimer Disease" = SEARCH 1 "Biobank terms" AND SEARCH 2 (2A, 2B, or 2C) "Neuro-urology terms" AND the following "Alzheimer Disease" terms:

<table>
<tr><td>Alzheimer[Title/Abstract]
OR "Alzheimer's"[Title/Abstract]
OR "Alzheimer-type"[Title/Abstract]
OR "Alzheimer Disease"[Mesh]</td><td rowspan="2">(73´861 results were found by entering these search terms only)</td></tr>
<tr><td>AND ((humans[Filter]) AND (2012/1/1:2022/12/31[pdat]))</td></tr>
</table>

SEARCH 4 "Spinal Cord Injury" = SEARCH 1 "Biobank terms" AND SEARCH 2 (2A, 2B, or 2C) "Neuro-urology terms" AND the following "Spinal Cord Injury" terms:

"Spinal Cord Injury"[Title/Abstract]<br>*OR "Spinal Cord Injuries"[Title/Abstract]*<br>*OR Paraplegia[Title/Abstract]*<br>*OR "Spinal Cord Injuries"[Mesh]*<br>*OR "Paraplegia"[Mesh]*	(22´059 results were found by entering these search terms only)
AND ((humans[Filter]) AND (2012/1/1:2022/12/31[pdat]))	

SEARCH 5 "Multiple Sclerosis" = SEARCH 1 "Biobank terms" AND SEARCH 2 (2A, 2B, or 2C) "Neuro-urology terms" AND the following "Multiple Sclerosis" terms:

"Multiple Sclerosis"[Title/Abstract]<br>*OR "Multiple Sclerosis"[Mesh]*	(36´172 results were found by entering these search terms only)
AND ((humans[Filter]) AND (2012/1/1:2022/12/31[pdat]))	

SEARCH 6 "Parkinson Disease" = SEARCH 1 "Biobank terms" AND SEARCH 2 (2A, 2B, or 2C) "Neuro-urology terms" AND the following "Parkinson Disease" terms:

"Parkinson Disease"[Title/Abstract]<br>*OR "Parkinsonian Disorders"[Title/Abstract]*<br>*OR "Parkinson Disease"[Mesh]*<br>*OR "Parkinsonian Disorders"[Mesh]*	(41´306 results were found by entering these search terms only)
AND ((humans[Filter]) AND (2012/1/1:2022/12/31[pdat]))	

SEARCH 7 "Stroke"= SEARCH 1 "Biobank terms" AND SEARCH 2 (2A, 2B, or 2C) "Neuro-urology terms" AND the following "Stroke" terms:

Stroke[Title/Abstract]<br>*OR Stroke[Mesh]*	(147´399 results were found by entering these search terms only)
AND ((humans[Filter]) AND (2012/1/1:2022/12/31[pdat]))	

SEARCH 8 "Diabetes"= SEARCH 1 "Biobank terms" AND SEARCH 2 (2A, 2B, or 2C) "Neuro-urology terms" AND the following "Diabetes" terms:

"Diabetic Neuropathies"[Title/Abstract]<br>*OR "Diabetic Nephropathy"[Title/Abstract]*<br>*OR "Diabetic Nephropathies"[Mesh]*<br>*OR "Diabetic Neuropathies"[Mesh]*	(20´290 results were found by entering these search terms only)
AND ((humans[Filter]) AND (2012/1/1:2022/12/31[pdat]))	

SEARCH 9 "Spina Bifida"= SEARCH 1 "Biobank terms" AND SEARCH 2 (2A, 2B, or 2C) "Neuro-urology terms" AND the following "Spina Bifida" terms:

"Spina Bifida"[Title/Abstract]<br>*OR "Spinal Dysraphism"[Title/Abstract]*<br>*OR "Spinal Dysraphism"[Mesh]*<br>*OR "Spina Bifida Cystica"[Mesh]*<br>*OR "Spina Bifida Occulta"[Mesh]*	(2'951 results were found by entering these search terms only)

AND ((humans[Filter]) AND (2012/1/1:2022/12/31[pdat]))	

Results:

The results of the different searches are depicted in Table S.2. Searches including SEARCH 2C are depicted in Figure 1 in the manuscript.

Table S.2. Results of the search strategies.

Search strategy		Number of results
	SEARCH 1 "Biobank terms"	1´247´877*
Searches incl. SEARCH 2A	SEARCH 2A "Neuro-urology terms", including neoplasms/cancer and generic terms not pointing directly to the neuro-urological field	419´068
	SEARCH 1 AND SEARCH 2A	105´862
	SEARCH 3 "Alzheimer Disease"	68
	SEARCH 4 "Spinal Cord Injury"	103
	SEARCH 5 "Multiple Sclerosis"	37
	SEARCH 6 "Parkinson Disease"	23
	SEARCH 7 "Stroke"	569
	SEARCH 8 "Diabetes"	3'027
	SEARCH 9 "Spina Bifida"	37
Searches incl. SEARCH 2B	SEARCH 2B "Neuro-urology terms", excluding neoplasms/cancer and including generic terms not pointing directly to the neuro-urological field	268´111
	SEARCH 1 AND SEARCH 2B	55´307
	SEARCH 3 "Alzheimer Disease"	53
	SEARCH 4 "Spinal Cord Injury"	86
	SEARCH 5 "Multiple Sclerosis"	32
	SEARCH 6 "Parkinson Disease"	18
	SEARCH 7 "Stroke"	542
	SEARCH 8 "Diabetes"	3'027
	SEARCH 9 "Spina Bifida"	36
Searches incl. SEARCH 2C	SEARCH 2C "Neuro-urology terms", excluding neoplasms/cancer and generic terms not pointing directly to the neuro-urological field	22´458
	SEARCH 1 AND SEARCH 2C	1´922 / 1'605**
	SEARCH 3 "Alzheimer Disease"	0
	SEARCH 4 "Spinal Cord Injury"	40
	SEARCH 5 "Multiple Sclerosis"	9

	SEARCH 6 "Parkinson Disease"	4
	SEARCH 7 "Stroke"	4
	SEARCH 8 "Diabetes"	3
	SEARCH 9 "Spina Bifida"	21

*of note, the bottom MSH term "Biomarkers" was responsible for more than half i.e. >1 mO of the search query outcome.

**Despite attempts to exclude reports in cancer patients, we found that in several of the 1'922 publications, prostate or bladder cancer was found to be a key word or the main topic of the study. We therefore made this search more stringent by adding the terms "NOT ("prostate cancer" OR "bladder cancer")", resulting in a total of 1'605 publications.

Conclusions:

1. Between 2012 and 2022, a total of 1´605 publications were found to include both neuro-urological search terms and biobanking search terms.
2. Of these 1´605 publications, a total of 81 were specifically linked to Spinal Cord Injury (40), Multiple Sclerosis (9), Parkinson's Disease (4), Stroke (4), Diabetes (3), and Spina Bifida (21).
